# Preliminary data on insulin sensitivity in non-obese prepubertal children affected by juvenile idiopathic arthritis

**DOI:** 10.1186/1546-0096-9-S1-P170

**Published:** 2011-09-14

**Authors:** Breda Luciana, Gaspari Stefania, De Sanctis Sara, Draghessi Antonella, Savino Alessandra, Scardapane Alessandra, Mohn Angelika, Chiarelli Francesco

## Background

Juvenile idiopathic arthritis (JIA) is one of the most common chronic inflammatory diseases in childhood. In adult patients with rheumatoid arthritis there is large body of evidence supporting the presence of insulin resistance (IR) mainly linked to the underlying inflammation; severity and duration of the disease influence insulin sensitivity and increase the risk of developing cardiovascular disease (CVD) and type 2 diabetes mellitus.

## Aim

To evaluate the presence of IR in non-obese prepubertal children affected by JIA, compared with healthy controls matched for age, sex and BMI.

## Methods

We enrolled 25 prepubertal non-obese children affected by JIA (according to ILAR criteria) and 50 matched healthy controls. JIA subjects at enrollment had at least 6 months of disease duration and showed active articular disease. Exclusion criteria included other autoimmune diseases or chronic inflammatory diseases, any medication that alters insulin action, family history of diabetes mellitus, obesity (BMI >95° percentile for age and sex) and systemic onset JIA. We collected data on age, sex, anthropometric values (height, weight, BMI, SDS-BMI) laboratory parameters (fasting glucose, fasting insulin, ESR, CRP). Disease activity score for JIA children was calculated using the JADAS-27 (*Juvenile Arthritis Disease Activity Score* based on a 27-joint count). Both JIA patients and controls underwent oral glucose tolerance test (OGTT) and insulin sensitivity indexes were calculated (HOMA-IR, QUICKI, WBISI, G/I).

## Results

JIA patients and controls matched for age, sex and anthropometric values. Both groups after OGTT did not show impaired glucose metabolism, although statistically significant difference was found in fasting insulin which was higher in the JIA group (14,8±27.6 *vs* 4.8±2.0; *p 0.011*). There also was a significant difference between the two groups in terms of insulin resistance indexes: HOMA-IR (1.55±1.21 in JIA *vs* 1.05±0.49 in controls, *p 0.011*), QUICKI (4.25±3.07 in JIA *vs* 1.17±0.16 in controls, p 0.0001) and WBISI (5.24±6.03 in JIA *vs* 10.87±3.59 in controls, *p 0.0001*). The AUC (area under the curve) for glucose and insulin showed no difference between JIA subjects and controls; after stratification of JIA patients in two groups (group 1= JADAS≤mean value; group 2=JADAS> mean value), a significant difference was found in terms of AUC for insulin among controls and JADAS group 2 (*p 0.03*). Differences in excursions of glucose and insulin during OGTT confirmed significant differences in fasting insulin and insulin after 30’ between all JIA patients and healthy controls.

## Conclusion

Preliminary data shown in our study allowed to detect initial insulin resistance among prepubertal non-obese JIA children compared to prepubertal healthy children. The main differences were found in the JIA subgroup with higher disease activity score, thus supporting the idea that inflammation plays a pivotal role in determining IR, although in presence of normal glucose metabolism. Early detection of metabolic alterations may represent an important tool in the monitoring of underlying inflammation and may be one of the targets in primary prevention of future CVD during adulthood.

